# Social Context Influences Resting Physiology in Dogs

**DOI:** 10.3390/ani10122214

**Published:** 2020-11-26

**Authors:** Kim Kortekaas, Kurt Kotrschal

**Affiliations:** 1 Department of Cognitive Biology, University of Vienna, Althanstrasse 14, 1090 Vienna, Austria; 2 Department of Behavioral Biology, University of Vienna, Althanstrasse 14, 1090 Vienna, Austria; kurt.kotrschal@univie.ac.at; 3 Wolf Science Center, Domestication Lab, Konrad-Lorenz Institute of Ethology, University of Veterinary Medicine, Savoyenstrasse 1a, 1160 Vienna, Austria

**Keywords:** alertness, conspecifics, dogs, domestication, heart rate, pack, resting, social context

## Abstract

**Simple Summary:**

Wolves became dogs over the past 35,000 years. It has been suggested that through domestication, the cooperative nature of wolves and their dependence on pack mates has been re-directed towards humans. This has also affected the social orientation of dogs towards conspecifics. In contrast to wolves, dogs in free-ranging packs are not monogamous and are less cooperative with their pack members. In a previous paper, we found that dogs resting isolated from their pack members were less relaxed/more alert than wolves in the same situation. As social context may affect such results, we replicated this study with pack-living and enclosure-kept dogs resting alone or close to pack members. Specifically, we measured heart rate and heart rate variability as physiological proxies of alertness. We found that dogs were less relaxed/more alert when resting alone than with pack members, but that this may be modulated by social status, i.e., high-ranking dogs being less relaxed than low-ranking individuals. We conclude that relaxation/alertness of dogs during rest depends on social context and that, as in wolves, conspecific pack members still have a role in this. This indicates that domestication has only partially re-directed social orientation in dogs from conspecific pack members to human partners.

**Abstract:**

Domestication has affected the social life of dogs. They seem to be less dependent on their pack members than wolves, potentially causing dogs to be more alert towards their environment, especially when resting. Such a response has been found in dogs resting alone compared to wolves in the same situation. However, as this may be influenced by social context, we compared alertness (i.e., degree of activation along the sleep–wake continuum—measured via cardiac parameters) of pack-living and enclosure-kept dogs in two conditions: (1) alone, and (2) with pack members, and in two states of activation: (1) inactive wakefulness, and (2) resting. We found that when dogs were resting alone, alertness was higher than when resting in the pack; individual alertness was potentially influenced by social rank. However, alertness was similar in the two conditions during inactive wakefulness. Thus, depending on social context, familiar conspecifics may still provide support in dogs; i.e., domestication has probably only partly shifted the social orientation of dogs from conspecifics to humans. We suggest that cardiac responses of dogs may be more flexible than those of wolves because of their adaptation to the more variable presence of humans and conspecifics in their environment.

## 1. Introduction

Some 35,000 years ago wolves became the first species to be domesticated [1,2,3,4,5]. Dogs (*Canis lupus familiaris)* share a common wolf ancestor with present day wolves (*Canis lupus*) [2,6] and are different from wolves with regard to their genetics, morphology, physiology, and social ecology [7,8,9,10]. Through domestication, humans became part of the dogs’ social environment [11], but dogs developed much flexibility in this, i.e., they can live as family pets, where food, shelter, and safety are provided, but also free-ranging, with their movements and actions not directly controlled by humans [12,13].

About 70–80% of dogs worldwide are free-ranging [14,15,16]. Their pack life differs in many ways from that of wolves. Generally, wolves live in relatively stable and cohesive family units around a monogamous pair, with age-graded hierarchies. Wolves rely on cooperation within their packs when raising offspring, hunting, and in territorial defense [17,18,19,20,21,22]. Free-ranging dogs can also live in packs and form long term social relationships [13,17,23], but they show much variation in their social organization. This probably depends on their genetics (i.e., genetic closeness to wolves; population origins: e.g., indigenous population, genetic mix of modern breeds; differences in relatedness to specific breeds; and relatedness among pack members), on local conditions (e.g., food availability, season), and on the extent of human influence [16,24,25]. Unlike wolves, pack dogs are generally promiscuous and rarely cooperate in the raising of offspring [26,27,28,29,30,31]. Pups are mainly raised by their mothers, but female-allocare (e.g., grandmother care) and paternal care have also been observed; males often provide care in the form of play and protection [15,30,31,32,33,34,35]. Like wolves, some studies found linear dominance hierarchies in dog packs (e.g., [25,36]), particularly based on age [13,37], while others did not (e.g., [12,33,38,39]). Hierarchies are generally also present in pet dogs and dogs in shelters [40,41,42].

While wolves rely heavily on each other when hunting large prey [43], dogs mainly rely on scavenging on human garbage, e.g., at dumps, or on food provisioned by humans [9,44,45]), and they typically forage solitarily [46]. When competing for food, agonistic interactions may be frequent [26,47,48]. However, it seems that intragroup aggression in free-ranging dogs is characterized by low intensity [37,48], possibly because high-ranking dogs monopolize food, while lower ranking dogs retreat [13,49,50,51]. Furthermore, in dog packs kept in enclosures, low rates of mild aggression between pack members were observed when compared to similarly raised wolves; however, when agonistic interactions occurred, they were often of high intensity [49].

Similar to wolves, many dog packs do defend their territories and food sources against other packs [26,32,47,48]. However, free-living dogs, in contrast to wolves, rarely engage in severe fighting when meeting [26,28,32,37,48,52,53]. Hence, dog packs seem to be socially less closed than wolf packs, which could be the basis for greater inter-pack tolerance in dogs as compared to wolves [25]. Overall, it seems that dogs might be less dependent on their pack members than wolves, especially with regard to foraging and pup-raising [9], but in general still coordinate their social life.

Survival and reproductive success of free-ranging dogs seems to depend much on their relationships with humans [54]. Hence, dogs may have adapted to living and interacting with humans by maintaining a generally higher alertness towards their environment than wolves, particularly at rest. This idea is supported by Kortekaas and Kotrschal [55], showing that individually resting pack dogs maintained greater alertness than resting wolves. Still, pack living dogs probably benefit from their pack members, even at rest. Adams and Johnson [56], for example, found non-synchronous sleep–wake cycles in situations where two or more dogs were sleeping together. They suggested that this might be a remnant of the evolutionary history of dogs, as the sleep cycles of ancestral wolves might have been asynchronous in the service of pack safety (i.e., the “many-eyes effect” [57]). Indeed, Majumder et al. [46] found that when resting, free-ranging dogs tend to form groups, but when foraging, they are in smaller subgroups or alone. Thus, alertness in dogs may still be shared amongst pack members when resting, allowing for decreased individual alertness in a pack situation as compared to resting alone.

Alertness to the environment, i.e., “the degree of activation along the sleep–wake continuum” [58,59], is physiologically regulated by the basal activity and reactivity of the two stress response systems, namely (1) the hypothalamic–pituitary–adrenocortical (HPA) system, which regulates the release of stress hormones (glucocorticoids such as cortisol) [60], and (2) the sympathetic–adrenal–medullary (SAM) system. Domestic guinea pigs, for example, showed less alertness to their physical environment than wild guinea pigs, while the reactivity of their HPA and SAM systems was reduced [61]. Hence, a reduction in the activity of these systems corresponds to a decrease in alertness. The stress systems are regulated by the sympathetic and parasympathetic branches of the autonomous nervous system (ANS); the ANS is primarily responsible for the maintenance of homeostasis within organisms and adapts its physiological response to changes in the internal environment of the body or the external environment. The sympathetic nervous system, for example, is activated when motor activity is required in order to protect and defend the body to external challenges, i.e., fight-or-flight mode [62]. Sleep is also regulated by the ANS [63]; from wakefulness to non-rapid eye movement (NREM) sleep parasympathetic activity normally increases, while sympathetic activity decreases. This suggests a shift from greater sympathetic activity towards vagal dominance from wakefulness to sleep. However, sympathetic activity appears to increase during REM sleep [64,65,66]. Hence, the transition from wakefulness to sleep is characterized by a decrease in responsiveness to environmental stressors [59]. Such modulation can be measured via different behavioral and physiological parameters, e.g., peeking (eyes open) rate and cardiac activity (i.e., heart rate (HR) and heart rate variability (HRV)) [55,59,66,67,68]. Peeking rate, for example, in green-winged teals decreased with increasing flock size [69], whereas disturbance by humans increased individual investment into vigilance [70]. Generally, in dogs, HR is lower and HRV is higher during sleep than when awake; when a dog is waking, HR increases and HRV decreases (i.e., increased sympathetic and decreased parasympathetic tone) [71,72].

In this study, we examined potential differences in alertness of pack-living and enclosure-kept dogs in two different conditions: (1) a dog resting alone, and (2) a dog resting in the presence of its pack members. As alertness is measured on a continuum between sleeping and being awake, we included two different states of activation on this continuum: (1) inactive wakefulness, and (2) resting (head down recumbency). We used cardiac parameters, i.e., HR and HRV, as physiological correlates of alertness. Following Kortekaas and Kotrschal [55], we used the respiratory sinus arrhythmia (i.e., regular HR increases during inhalation and decreases during exhalation [73]) to distinguish periods of deep relaxation during resting. We have shown previously that when resting alone, dogs show a greater alertness than equally raised and kept wolves [55]. In the current study, we hypothesize that dogs will benefit from the presence of their pack members, allowing lower alertness at rest in the pack condition than in the alone condition (i.e., lower HR, higher HRV).

## 2. Materials and Methods

### 2.1. Ethical Approval

The dogs were kept at the Wolf Science Center situated in the Ernstbrunn Game Park (License No. AT00012014) in Austria (www.wolfscience.at; 48°32′49.5′′ N, 16°20′51.5′′ E). They were kept according to the requirements for the management and handling of animals defined by the Austrian Federal Act on the Protection of Animals (Animal Protection Act—TSchG, BGBl. I Nr. 118/2004). Dogs voluntarily chose to participate in test sessions; in the rare case that a subject was not motived to participate, the test session was cancelled and repeated on another day, which also standardized the motivation of the participating dogs. As the methods used were non-invasive, we did not need special permission for the execution of this study conforming to the Austrian Animal Experiments Act (BGBl. I Nr. 114/2012, Tierversuchsgesetz 2012—TVG 2012).

### 2.2. Subjects

Data were collected from 6 individual male dogs, *Canis lupus familiaris* (Table 1). These subjects were chosen because they were the most stable during training and testing with regard to the heart rate (HR) monitor application. For this reason and because two sexes in the sample would have constrained model stability (below), we decided not to include females in this study. The dogs were 2.7–3.8 (mean ± SD = 3.30 ± 0.41) years of age when tested. The dogs were socialized and hand-raised by humans from when they were 10 days old. At the age of 5 months, our dogs were introduced into pre-existing packs with adult pack members—see also Range and Virányi [10]. The dogs stayed in stable packs of different sizes (2–3 animals) in natural enclosures ranging from 2000 to 8000 m^2^ in size, which included trees and bushes, and objects such as branches and stones. The enclosures also included heated shelters for the dogs. The dogs had ad libitum access to water—also during testing—and were fed daily with Medium Adult dry food from Royal Canin^®^. Regularly, small chunks of chicken, deer, and/or rabbit were added to the dry food as well. During their daily training sessions (based on positive reinforcement methods) or when being tested (on average 4 times a week) the dogs were rewarded with small portions (±1 cm^2^) of beef, sausage, or German Shepherd dry food from Royal Canin^®^.

### 2.3. Data Collection

#### 2.3.1. Heart Rate Measurements

The dogs’ HR and HR variability (HRV) responses were measured using the Polar^®^ RS800CX HR monitor [55]. The Polar^®^ HR monitor was designed for humans, but is increasingly used in animal studies, including in dogs [74] (see for example [75,76,77,78,79]). This HR monitor was also validated against the electrocardiogram (ECG) for dogs [74,80,81]. The HRV parameter used in this study was the root mean square of successive differences (RMSSD), a parameter frequently used to analyze short-term HRV; see also von Borell et al. [82].

The HR monitor consisted of three separate parts: (1) a chest belt with electrodes, (2) a WearLink^®^ W.I.N.D transmitter for wireless data transmission (connected to a chest belt during experimental sessions), and (3) a data logger (watch). The chest belt was tied around the chest of the animals, just behind the shoulder blades. We made sure that the electrodes were placed on the left side, i.e., over the heart, and the fur between skin and electrodes was wetted with 70% ethanol in order to improve the transmission of the HR signal to the electrodes. The watch was attached to a collar that was tied around the neck of the dogs.

#### 2.3.2. Experimental Setup

We measured the HR and HRV of the dogs in two different conditions: (1) alone, where the focal animal was visually separated from its pack members, and (2) in a pack, where the focal animal was together with all its pack members. Following Kortekaas and Kotrschal [55], we investigated the HR and HRV of the dogs in two different states of activation: (1) inactive wakefulness, and (2) resting. These two states were behaviorally defined by Kortekaas and Kotrschal [55] as follows:

1. “Inactive wakefulness: body touching the ground either with caudal, dorsal, or lateral side. Position of the paws varies, e.g., folded (under body) or stretched out. Head is in an upward position and can be moved around. Eyes are open, but increased blinking can occur.”
2. “Resting: body touching the ground either with caudal, dorsal, or lateral side. Position of the paws varies, e.g., folded (under body) or stretched out. Head is in a downward position, either lying on paws, ground, or tucked under the body. Eyes are generally closed, but might be opened and closed again (peeking). Parts of the body occasionally twitching.”

### 2.4. Procedure

We collected the data in the summer of 2013 (with 1 observation day added in November) during the day (between 09:00 and 18:00); we tried to collect most of the data in periods of the day when the dogs were most likely to rest anyway (typically between 12:00 and 14:00 [83]).

A test session started with the shifting of the focal subject from the enclosure where it was kept into an airlock. Here, the HR monitor was placed on the subject. The shifting of animals and the application of the HR monitor are both procedures at the Wolf Science Center which were taught through positive reinforcement training and performed with the full cooperation of the animals. Hence, shifting and all other manipulations in this study were routine procedures for the dogs. After the HR monitor was applied to the focal individual, depending on the condition, the animal was either shifted to an enclosure without other pack members (alone condition) or shifted back into its home enclosure with its pack members (pack condition) for the period of testing. Generally, test sessions lasted about 1 h and were recorded on video by a human experimenter. The human experimenter and focal subject were separated by a fence, and the human did not interact with the dog(s).

It has been suggested that the testing environment and the duration of housing there might influence cortisol and arousal levels in dogs [84,85]. However, as the dogs in the Wolf Science Center are separated daily from their pack members for research and management purposes and are rotated weekly (with their pack members) into a variety of enclosures, we do not think the shifting of the dogs for the HR monitor handling or change of enclosure in the alone condition influenced the cardiac output of the dogs, because they are well habituated to these situations.

### 2.5. Data Selection

Data were selected using the procedure of Kortekaas and Kotrschal [55]. We started by scanning all the HR data files for patterns consistent with that of a respiratory sinus arrhythmia (RSA). Only sections that displayed a regular RSA pattern were selected, but only if they were longer than 1 min [71]. The video recordings of these HR parts were then checked to see if the behavior of the subjects in the videos was consistent with that of the behavioral definition for the state category “resting”. In the pack condition, we only selected the HR parts where pack members were resting at least one body length away from each other (which occurred in most instances anyway). The longest RSA collected from one animal lasted only 2 min; hence, all HR parts used for analyses were also limited to 2 min—von Borell et al. [82] recommend the recording lengths of HR(V) data to be of equal length for analyses. Hereafter, in contrast to Kortekaas and Kotrschal [55], we investigated the videos of all the HR files for the presence of behavior consistent with that of the behavioral definition for the state category “inactive wakefulness”. If these parts were 2 min long, they were selected for analyses. If multiple usable HR parts in one HR file were found, we randomly selected one, i.e., drawing one from the available HR parts, for further analyses. If the HR parts selected contained errors, they were corrected using the AVEC-method (see Schöberl et al. [86] for details); the confidence level used for outliers was set to 75%.

In total, 70 data points (Data S1) were collected from six dogs. We collected a minimum of 1 recording and a maximum of 4 repetitions per state, per condition, and per subject (in total 96 data points could have been collected). We excluded 18 data points, however, because (1) we did not find a RSA pattern in the HR data, and/or (2) the subjects did not display the behaviors required for inclusion (i.e., inactive wakefulness/resting), or (3) the observed RSA patterns or continuous behavior periods observed were too short (less than 1 min) to be included in the analyses. For the inactive wakefulness state in the pack condition, we excluded 3 data points. For the resting state in the alone condition, 11 were excluded, while only 4 were excluded in the pack condition. In addition, at the start of data collection, one of the dogs (Hakima) was taken out of its pack permanently, because of behavioral incompatibility with its pack members; consequently, for Hakima no pack data (i.e., 8 data points) could be recorded.

To increase the sample size of the data analyzed, some of the HR files collected for the Kortekaas and Kotrschal [55] study were again included in this study, i.e., some of the HR files of dogs in the alone condition. As often more than one usable HR part was found in these HR data files, we reprocessed the data in these files, but used other HR parts for the analyses in this study. Hence, only 8 of the data points integrated in this study were reused; however, as the HR data parts in this study were longer than that in the Kortekaas and Kotrschal [55] study, i.e., 40 s longer, new mean HRs and HRVs were also calculated for these 8 data points.

### 2.6. Statistical Analyses

To investigate the effects of state (inactive wakefulness or resting) and condition (alone or pack) on the cardiac activity of dogs, we carried out two separate linear mixed effect models (LMM) [87] with (1) mean HR and (2) HRV—expressed as the RMSSD—as their response variables. These models were completely similar with regard to their fixed and random effect structures. State, condition, and their interaction were added as fixed factors. We considered the interaction the key term in the model. To control for the effects of temperature (i.e., indicator of environmental conditions), body weight, and number of pack members (NPM), these factors were added as fixed effects as well. However, they were z-transformed to a mean of zero and a standard deviation of one to make model estimates more easily interpretable and to make it more likely for the models to converge [88].

As multiple measurements on the same individuals were collected, we added individual as a random intercept. Additionally, as multiple measurements (i.e., one data point of inactive wakefulness and one of resting) were often collected from one animal on the same day (62.79% of the cases), we combined individual and day into a grouping factor (i.e., day nested into individual), which was also added as a random intercept. To avoid overconfident estimates of fixed effects and to reduce the type I error rate, we included random slopes [89,90]. More specifically, random slopes of state, condition, and temperature were added within individual. For this purpose, state and condition were manually dummy-coded and then centered.

Because many of the correlations between the random intercepts and slopes for the maximum HR and HRV models were estimated to be essentially one, we removed them from the models, as the models were probably too complex to estimate the random effects reliably [91]. This only led to a small decrease in the model fit (log-likelihood or logLik) for both the HR and HRV models (HR model with correlations: logLik = −257.65; HR model without correlations: logLik = −260.89; HRV model with correlations: logLik = −424.54; HRV model without correlations: logLik = −427.19).

To evaluate whether the HR and HRV models violated the assumptions of normality and homogeneity of the residuals, we inspected qq-plots of their residuals and scatterplots of their residuals plotted against the fitted values. For both models, the assumptions did not seem to be violated. We also checked if there were problems with collinearity among the fixed effects. Hence, we fitted a standard linear model but excluded the interaction of state and condition along with the random effects. We then used the vif function within the R-package car (Version 3.0-3 [92]) to create the variance inflation factors (VIFs) [93]. We concluded that collinearity among the predictors was not a problem (maximum VIF = 1.12) [94]. Finally, we evaluated model stability by comparing full model estimates with those obtained by models in which the levels of the random effects were excluded one at a time [95]. For both the HR and HRV model, the fixed factors were sufficiently stable, although most random effects were not.

With the use of a likelihood ratio test [96] (argument “test” set to “Chisq”), we assessed the overall effect of state, condition, and their interaction by comparing the full model with that of a null model containing only temperature, body weight, and NPM as well as the random effects [97]. In addition, likelihood ratio tests (R function drop1 with argument “test” set to “Chisq”) were also used to investigate the influence of the individual fixed effects by comparing the full model with reduced models in which the individual fixed effects were excluded one at a time [89].

The statistical analyses were carried out in R (Version 3.6.1 [98]), using the function lmer of the package lme4 (Version 1.1-21 [99]) with the optimizer “bobyqa” for the RMSSD null model. Model stability was assessed with a function kindly provided by Roger Mundry. We obtained our confidence intervals by bootstrapping over the random effects using the R function bootMer of the package lme4 and using 1000 parametric bootstraps (argument “use.u” set to TRUE; see also Data S2 for the R script of the HR and HRV analyses).

## 3. Results

### 3.1. Heart Rate

**Figure 1:**
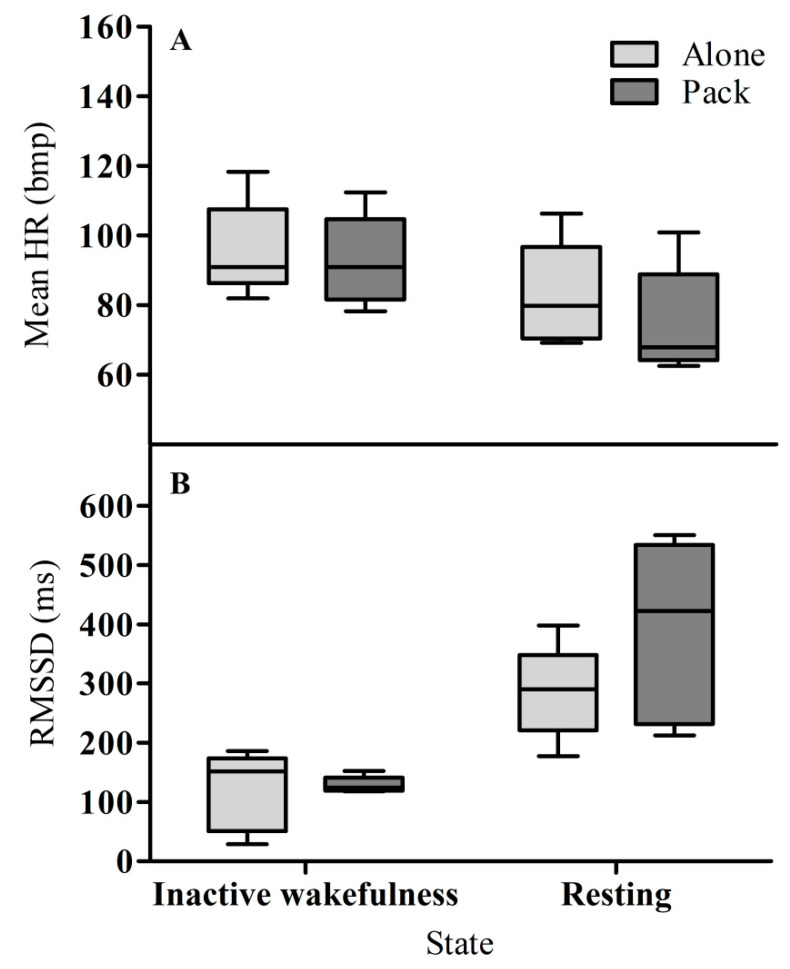
Mean HR (**A**) and RMSSD (**B**) of dogs during inactive wakefulness and resting in the alone and pack conditions. Boxes encompass the interval between the 25th and 75th percentiles, the horizontal line represents the median, and whiskers give the 5 and 95 percentiles.

Overall, the factors state and condition as well as their interaction had an influence on the dogs’ mean heart rate (HR; full–null model comparison, LRT: χ2 = 17.493, df = 3, *p* < 0.001). HR during inactive wakefulness was higher than when resting, regardless of the social condition. Additionally, the HR during inactive wakefulness did not seem to depend on the social context; i.e., during inactive wakefulness the dogs showed similar HRs in the alone and pack condition. However, when resting, HR was lower in the pack condition than in the alone condition (test of interaction between state and condition, LRT: χ2 = 3.856, df = 1, *p* = 0.050; Table 2; Figure 1A; Figure S1A,B). None of the control factors (temperature, body weight, and number of pack members) had a significant effect on the HR (Table 2).

### 3.2. Heart Rate Variability (RMSSD)

Overall, the full model was significant as compared to the null model, i.e., state, condition, and/or their interaction seemed to affect heart rate variability (HRV), expressed as the RMSSD (full–null model comparison, LRT: χ2 = 17.513, df = 3, *p* < 0.001). The RMSSD during inactive wakefulness was lower than when resting, irrespective of the social condition. Additionally, during inactive wakefulness, the RMSSD was similar in the alone and pack conditions. However, the RMSSD of dogs resting in the pack condition was higher than when resting alone (test of interaction between state and condition, LRT: χ2 = 3.930, df = 1, *p* = 0.047; Table 3; Figure 1B; Figure S1C,D). Interestingly, this group effect seemed to be mainly driven by two individuals (i.e., Maisha and Meru; Figure S1D). None of the control factors (temperature, body weight, and number of pack members) had a significant effect on the HRV, although the RMSSD seemed to decrease with increasing body weight (Table 3).

## 4. Discussion

During inactive wakefulness, irrespective of social context, dogs had a higher heart rate (HR), lower heart rate variability (HRV; i.e., showed higher sympathetic activation), and were more alert than when resting. This aligns with results that resting individuals are less aware and less responsive to their environment than when awake [55,71,100]. More interestingly, we found that when the dogs were resting in the alone condition, their HR was higher and HRV was lower than in the pack condition. Thus, when dogs were resting with their pack members, they showed the lowest levels of alertness.

During inactive wakefulness, however, the social isolation of the dogs from their pack members did not result in differences in the cardiac output between the two conditions. We suggest that dogs may be quite flexible in their response to their (social) environment. Being alone might raise an individual’s attention towards their non-social environment, while being with group members may decrease and distribute attentiveness, but may also increase social stress. Hence, the stress response systems of dogs are probably tuned in an adaptive way in order to respond appropriately to the different challenges of their (social) environment. In this case, this may explain the equally high cardiac activity of the dogs in both conditions. Our results seem to be in line with studies using cortisol as a measure of arousal that compared dogs living solitarily or in pairs/groups [84,101] and pair-housed dogs being temporarily separated [102].

On the other hand, the cardiac activity of the resting dogs was lower with conspecifics than alone. In this case, the dogs might have found the presence of their pack members reassuring; the functional increase of safety of resting in a pack versus resting alone may show mentally via lower arousal levels. Similarly, in resting prairie voles (*Microtus ochrogaster*), social isolation increased HR and decreased HRV [103], which was also the case for the HR of mice housed alone versus housed in pairs [104]. Being with partners may also socially support and mentally buffer when challenged. For example, in single-dog households, individuals showed a greater cortisol response to a simulated thunderstorm than dogs living with conspecifics; interestingly, the baseline cortisol levels of the dogs living in multi-dog households were slightly higher than those from the solitarily living dogs, potentially reflecting the social demands of group living. Actually, the presence of conspecifics seemed to be more important in this test situation than the presence of the owners. This indicates the still potentially important role of conspecifics in the dogs’ responses to their environment [105].

We also noticed that resting behavior in the alone condition was harder to observe than in the pack condition (i.e., we were 1.6 times more likely to observe resting behavior in the pack condition than in the alone condition). Hence, it seems that individuals rested more readily, because they were more relaxed when in the pack; this was also found in mice [104,106]. Actually, high flexibility with regard to sleeping patterns in dogs has already been suggested, allowing dogs to adapt their sleep–wake cycles to those of their humans [107,108,109], but possibly also to the presence/absence of conspecifics.

With regard to HRV, the relaxation effect in the pack condition seems to be driven mainly by two sub-dominant animals, i.e., Meru and Maisha (Table 1; Figure S1D). Their HRV was higher than that of the other subjects. It has been suggested that a relationship between social status and the hypothalamic–pituitary–adrenocortical (HPA) stress response system exists. Elevated glucocorticoid levels can be the result of subordination or the price of being dominant [60,110,111,112,113]. The dominance hierarchies in our captive dog packs seem to be steeper than those of our wolves. Dominant animals were observed to show more agonistic behavior than subordinate animals, at least in a feeding context [51], and at an absence of reconciliation behavior [49]. Hence, it is possible that dominant dogs have to invest more into social attentiveness than subordinate dogs. As the HPA and the sympathetic–adrenal–medullary (SAM) systems play important roles in the response of animals to their social environment, dominant animals could also have an upregulated basal SAM system as compared to subordinate animals.

It is an ongoing debate whether the allometric relationship between body size and HR in dogs may be inversely proportional, i.e., a Chihuahua should have a higher HR than a Great Dane. Hence, body size could have a considerate influence on HR in dogs. In our dogs, differences in body mass were considerably minor (15.45–34.60 kg), but still could have an effect on the parameters we measured. However, only a single study found such an effect of body size on HR in dogs [114], whereas a number of other studies have not [55,115,116,117,118]. This supports our present results that there was no influence of body size, although the RMSSD did show a trend towards decreasing with increasing body mass. Finally, we realize that our sample size is relatively small, but we are confident that our results provide an exciting base for the further exploration of the intraspecific social orientation of dogs.

## 5. Conclusions

It seems that our pack living dogs relax more deeply in the presence of their pack members than when resting alone, but there could be inter-individual variation depending on social status. We conclude that dog alertness during rest depends on social context and that conspecific pack members still have a role in this. This indicates that domestication has only partially re-directed social orientation in dogs from conspecific pack members to human partners and may have produced a more varied pattern in dogs as compared to wolves than is generally acknowledged by the mainstream domestication hypotheses.

## Figures and Tables

**Table 1:** Sex, pack membership, number of pack members (NPM), rank estimation (high or low) of social hierarchy, average body weight, average age when tested, and kinship of dog subjects.

Dog	Sex	Pack #	NPM	Rank ^1^	Body Weight (kg)	Age (Year)
Asali	♂	1	1	High	31.04	2.9
Hakima	♂	-	-	-	15.45	2.8
Kilio	♂	2	1	High	26.95	3.7 ^2^
Maisha	♂	3	2	Low	24.44	3.7 ^2^
Meru	♂	4 (5)	1	Low	34.60	2.8
Rafiki	♂	3	2	High	19.64	3.7

^1^ Observations of social interactions between pack members allowed for an estimation of the hierarchy of the dogs in their packs (see also [49]). ^2^ Siblings.

**Table 2:** Results of the heart rate (HR) model (estimates, standard errors, and confidence intervals (CIs)), results of the likelihood ratio tests (χ2, df, and p), and the estimates obtained for model stability (min and max).

Term	Est	SE	Lower CI	Upper CI	χ2	df	p	Min	Max
Intercept	96.228	4.772	87.128	105.321			^1^	89.688	102.114
State ^2^	−11.461	2.538	−16.255	−6.749			^1^	−13.294	−9.760
Condition ^3^	−5.262	6.254	−17.070	7.297			^1^	−11.595	−0.604
Temperature ^4^	−0.481	1.642	−3.638	2.560	0.081	1	0.776	−1.317	−0.122
Weight ^5^	3.469	3.651	−4.214	11.553	0.884	1	0.347	−1.805	7.959
NPM ^6^	1.943	3.904	−5.655	9.472	0.243	1	0.622	−3.399	7.434
State:Condition	−5.307	2.624	−10.533	−0.360	3.856	1	**0.050 ^7^**	−7.622	−3.715

^1^ Not indicated because of having very limited interpretation. ^2^ Dummy coded with Awake being the reference category. ^3^ Dummy coded with Alone being the reference category. ^4^ Z-transformed to a mean of 0 and standard deviation of 1; original mean and sd were 22.678 and 4.708 °C, respectively. ^5^ Z-transformed to a mean of 0 and standard deviation of 1; original mean and sd were 25.788 and 5.982 kg, respectively. ^6^ Z-transformed to a mean of 0 and standard deviation of 1; original mean and sd were 1.288 and 0.640 pack members, respectively. ^7^ Bold value indicates statistical significance.

**Table 3:** Results of the RMSSD model (estimates, standard errors, and confidence intervals (CIs)), results of the likelihood ratio tests (χ2, df, and p), and the estimates obtained for model stability (min and max).

Term	Est	SE	Lower CI	Upper CI	χ2	df	p	Min	Max
Intercept	132.082	24.989	85.355	175.707			^1^	107.514	139.409
State ^2^	142.074	40.145	62.199	216.346			^1^	128.355	166.965
Condition ^3^	6.677	36.688	−65.434	78.870			^1^	−14.192	27.809
Temperature ^4^	−26.511	15.030	−56.345	1.782	2.274	1	0.132	−36.163	−16.589
Weight ^5^	−31.192	15.403	−63.686	−3.041	3.799	1	0.051	−43.397	15.742
NPM ^6^	26.870	15.578	−5.351	57.150	2.827	1	0.093	18.702	87.269
State:Condition	94.399	44.382	8.527	185.034	3.930	1	**0.047 ^7^**	47.253	133.593

^1^ Not indicated because of having very limited interpretation. ^2^ Dummy coded with Awake being the reference category. ^3^ Dummy coded with Alone being the reference category. ^4^ Z-transformed to a means of 0 and standard deviation of 1; original mean and sd were 22.678 and 4.708 °C, respectively. ^5^ Z-transformed to a means of 0 and standard deviation of 1; original mean and sd were 25.788 and 5.982 kg, respectively. ^6^ Z-transformed to a mean of 0 and standard deviation of 1; original mean and sd were 1.288 and 0.640 pack members, respectively. ^7^ Bold value indicates statistical significance.

## Supplementary Materials

The following are available online at https://www.mdpi.com/2076-2615/10/12/2214/s1, Figure S1: Mean HR and RMSSD of individual dogs during inactive wakefulness and resting in the alone and pack conditions, Data S1: Dataset, Data S2: R Script of HR and HRV analyses.
