# Supplementary material for: Social Context Influences Resting Physiology in Dogs
Source: Animals (Basel). 2020 Nov 26;10(12):2214. doi: 10.3390/ani10122214 (PMC7760264; doi:10.3390/ani10122214)
Supplement: Supplementary file 1 [file animals-10-02214-s001.zip › Kortekaas_Kotrschal_Data S2.docx]

HEART RATE

# Used library(ies)

library(car)

library(lme4)

# Functions (provided by Roger Mundry)

source("/.../diagnostic_fcns.r")

source("/.../glmm_stability.r")

source("/.../boot_glmm.r")

# Used file (xdata)

summary(xdata)

# How many observations do we have per subject and per day?

xx = aggregate(x=1:nrow(xdata), by=xdata[, c("Animal", "Date")],FUN=length)

sum(xx$x>1)

mean(xx$x>1)

# Include a random effect combining individual and date

xdata$An.Date = as.factor(apply(X=xdata[, c("Animal", "Date")], MARGIN=1, FUN=paste, collapse="_"))

# DATA PREP

# Range

range(xdata$HR)

range(xdata$Temperature)

range(xdata$Weight)

range(xdata$NPM)

# Distribution of variables

hist(xdata$HR)

hist(xdata$Temperature)

hist(xdata$Weight)

# Random slopes

source("/.../diagnostic_fcns.r")

xx.fe.re = fe.re.tab(fe.model = "HR ~ Condition * State + Temperature + Weight + NPM",

re = "(1 | Animal) + (1 | An.Date)", data = xdata)

xx.fe.re$summary[1]

xx.fe.re$summary[2]

xx.fe.re$summary[3:4]

xx.fe.re$summary[5:6]

xx.fe.re$summary[7]

xx.fe.re$summary[8]

xx.fe.re$summary[9:10]

xx.fe.re$summary[11:12]

xx.fe.re$summary[13:14]

xx.fe.re$summary[15:16]

xx.fe.re$summary[17]

xx.fe.re$summary[18]

# MODEL

# Extract dummy coded factors

str(xx.fe.re$data)

t.data = xx.fe.re$data

# Center the dummy coded factors

t.data$Condition.Pack = t.data$Condition.Pack - mean(t.data$Condition.Pack)

t.data$State.Resting = t.data$State.Resting - mean(t.data$State.Resting)

# Z-transform

t.data$z.Temperature = as.vector(scale(t.data$Temperature))

t.data$z.Weight = as.vector(scale(t.data$Weight))

t.data$z.NPM = as.vector(scale(t.data$NPM))

full = lmer(HR ~ Condition * State + z.Temperature + z.Weight + z.NPM

+ (1 + Condition.Pack + State.Resting + z.Temperature | Animal)

+ (1 | An.Date),

data = t.data, REML=FALSE)

summary(full)$varcor

# Model is too complex to estimate all random effects reliably; remove correlations among random intercepts and slopes

full.red = lmer(HR ~ Condition * State + z.Temperature + z.Weight + z.NPM

+ (1 + Condition.Pack + State.Resting + z.Temperature || Animal)

+ (1 | An.Date),

data = t.data, REML=FALSE)

summary(full.red)$varcor

# How much difference does it make from old to new model?

ll.old = logLik(full)

ll.new = logLik(full.red)

ll.old

ll.new

# Removal of correlations only lead to a small decrease of the maximum likelihood: use model full.red

# ASSUMPTIONS

# Normality + homogeneity

diagnostics.plot(full.red, size.fac = 2)

ranef.diagn.plot(full.red)

# Collinearity

xx = lm(HR ~ Condition + State + z.Temperature + z.Weight + z.NPM, data = t.data)

vif(xx)

# Stability

source("/.../glmm_stability.r")

full.stab = glmm.model.stab(model.res = full.red, contr=NULL, para=F, data = NULL)

# Evaluate model stability

head(full.stab$detailed, 4)

table(full.stab$detailed$warnings)

round(full.stab$summary[, -1], 3)

# Model stability: plotting results

is.re=grepl(x=rownames(full.stab$summary), pattern="@")

m.stab.plot(full.stab$summary[!is.re, -1])

m.stab.plot(full.stab$summary[is.re, -1])

# INFERENCE FIXED EFFECTS

# Null model

null = lmer(HR ~ z.Temperature + z.Weight + z.NPM

+ (1 + Condition.Pack + State.Resting + z.Temperature || Animal)

+ (1 | An.Date),

data = t.data, REML=FALSE)

as.data.frame(anova(null, full.red, test="Chisq"))

tests=as.data.frame(drop1(full.red, test="Chisq"))

round(tests, 3)

summary(full.red)

# Confidence intervals

source("/.../boot_glmm.r")

boot.full = boot.glmm.pred(model.res=full.red, excl.warnings=F,nboots=1000, para=T, n.cores=2,

resol=1000, level=0.95)

round(boot.full$ci.estimates, 3)

m.stab.plot(boot.full$ci.estimates)

HEART RATE VARIABILITY (RMSSD)

# Used library(ies)

library(car)

library(lme4)

# Functions (provided by Roger Mundry)

source("/.../diagnostic_fcns.r")

source("/.../glmm_stability.r")

source("/.../boot_glmm.r")

# Used file (xdata)

summary(xdata)

# How many observations do we have per subject and per day?

xx = aggregate(x=1:nrow(xdata), by=xdata[, c("Animal", "Date")],FUN=length)

sum(xx$x>1)

mean(xx$x>1)

# Include a random effect combining individual and date

xdata$An.Date = as.factor(apply(X=xdata[, c("Animal", "Date")], MARGIN=1, FUN=paste, collapse="_"))

# DATA PREP

# Range

range(xdata$RMSSD)

range(xdata$Temperature)

range(xdata$Weight)

range(xdata$NPM)

# Distribution of variables

hist(xdata$RMSSD)

hist(xdata$Temperature)

hist(xdata$Weight)

# Random slopes

source("/.../diagnostic_fcns.r")

xx.fe.re = fe.re.tab(fe.model = "RMSSD ~ Condition * State + Temperature + Weight + NPM",

re = "(1|Animal) + (1|An.Date)", data = xdata)

xx.fe.re$summary[1]

xx.fe.re$summary[2]

xx.fe.re$summary[3:4]

xx.fe.re$summary[5:6]

xx.fe.re$summary[7]

xx.fe.re$summary[8]

xx.fe.re$summary[9:10]

xx.fe.re$summary[11:12]

xx.fe.re$summary[13:14]

xx.fe.re$summary[15:16]

xx.fe.re$summary[17]

xx.fe.re$summary[18]

# MODEL

# Extract dummy coded factors

str(xx.fe.re$data)

t.data = xx.fe.re$data

# Center the dummy coded factors

t.data$Condition.Pack = t.data$Condition.Pack - mean(t.data$Condition.Pack)

t.data$State.Resting = t.data$State.Resting - mean(t.data$State.Resting)

# Z-transform

t.data$z.Temperature = as.vector(scale(t.data$Temperature))

t.data$z.Weight = as.vector(scale(t.data$Weight))

t.data$z.NPM = as.vector(scale(t.data$NPM))

full = lmer(RMSSD ~ Condition * State + z.Temperature + z.Weight + z.NPM

+ (1 + Condition.Pack + State.Resting + z.Temperature | Animal)

+ (1 | An.Date),

data = t.data, REML=FALSE)

summary(full)$varcor

# Model is too complex to estimate all random effects reliably; remove correlations among random intercepts and slopes

full.red = lmer(RMSSD ~ Condition * State + z.Temperature + z.Weight + z.NPM

+ (1 + Condition.Pack + State.Resting + z.Temperature || Animal)

+ (1 | An.Date),

data = t.data, REML=FALSE)

summary(full.red)$varcor

# How much difference does it make from old to new model?

ll.old = logLik(full)

ll.new = logLik(full.red)

ll.old

ll.new

# Removal of correlations only lead to a small decrease of the maximum likelihood: use model full.red

# ASSUMPTIONS

# Normality + homogeneity

diagnostics.plot(full.red, size.fac = 2)

ranef.diagn.plot(full.red)

# Collinearity

xx = lm(RMSSD ~ Condition + State + z.Temperature + z.Weight + z.NPM, data = t.data)

vif(xx)

# Stability

source("/.../glmm_stability.r")

full.stab = glmm.model.stab(model.res = full.red, contr=NULL, para=F, data = NULL)

# Evaluate model stability

head(full.stab$detailed, 4)

table(full.stab$detailed$warnings)

round(full.stab$summary[, -1], 3)

# Model stability: plotting results

is.re=grepl(x=rownames(full.stab$summary), pattern="@")

m.stab.plot(full.stab$summary[!is.re, -1])

m.stab.plot(full.stab$summary[is.re, -1])

# INFERENCE FIXED EFFECTS

# Null model

null = lmer(RMSSD ~ z.Temperature + z.Weight + z.NPM

+ (1 + Condition.Pack + State.Resting + z.Temperature || Animal)

+ (1 | An.Date),

data = t.data, REML=FALSE)

# Model failed to converge so add control

contr=lmerControl(optimizer="bobyqa", optCtrl=list(maxfun=10000))

null = lmer(RMSSD ~ z.Temperature + z.Weight + z.NPM

+ (1 + Condition.Pack + State.Resting + z.Temperature || Animal)

+ (1 | An.Date),

data = t.data, REML=FALSE, control = contr)

as.data.frame(anova(null, full.red, test="Chisq"))

tests=as.data.frame(drop1(full.red, test="Chisq"))

round(tests, 3)

summary(full.red)

# Confidence intervals

source("/.../boot_glmm.r")

boot.full = boot.glmm.pred(model.res=full.red, excl.warnings=F,nboots=1000, para=T, n.cores=2,

resol=1000, level=0.95)

round(boot.full$ci.estimates, 3)

m.stab.plot(boot.full$ci.estimates)
