# Supplementary material for: Social Context Influences Resting Physiology in Dogs
Source: Animals (Basel). 2020 Nov 26;10(12):2214. doi: 10.3390/ani10122214 (PMC7760264; doi:10.3390/ani10122214)
Supplement: Supplementary file 1 [file animals-10-02214-s001.zip › Kortekaas_Kotrschal_Figure S1.docx]

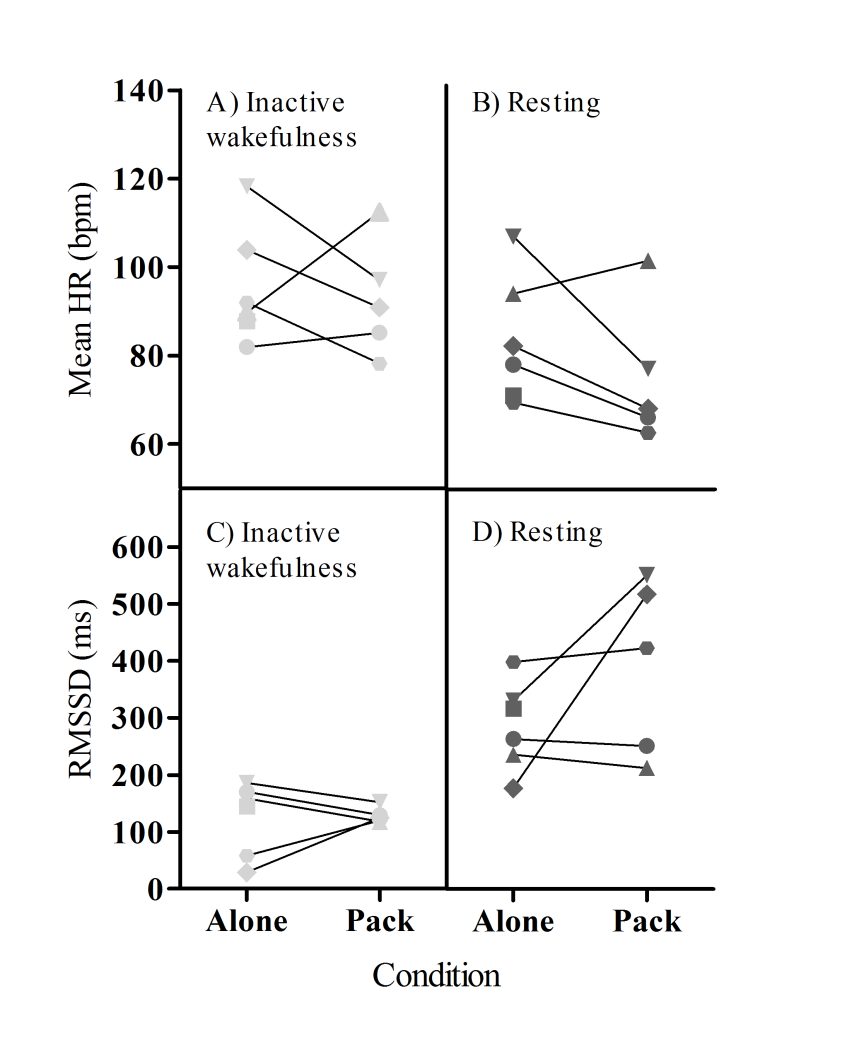


**Figure S1.** Mean HR of individual dogs during inactive wakefulness (A) and resting (B) and RMSSD of individual dogs during inactive wakefulness (C) and resting (D) in the alone and pack conditions. Boxes encompass the interval between the 25^th^ and 75^th^ percentiles, the horizontal line represents the median, and whiskers give the 5 and 95 percentiles.
